# A subduction influence on ocean ridge basalts outside the Pacific subduction shield

**DOI:** 10.1038/s41467-021-25027-2

**Published:** 2021-08-06

**Authors:** A. Y. Yang, C. H. Langmuir, Y. Cai, P. Michael, S. L. Goldstein, Z. Chen

**Affiliations:** 1 State Key Laboratory of Isotope Geochemistry, Guangzhou Institute of Geochemistry, Chinese Academy of Sciences, Guangzhou, China; 2 Key Laboratory of Ocean and Marginal Sea Geology, Guangzhou Institute of Geochemistry, Chinese Academy of Sciences, Guangzhou, China; 3 CAS Center for Excellence in Deep Earth Science, Guangzhou, China; 4 Department of Earth and Planetary Sciences, Harvard University, Cambridge, MA USA; 5 Lamont-Doherty Earth Observatory, Columbia University, New York, NY USA; 6 University of Tulsa, Tulsa, OK USA

**Keywords:** Geochemistry, Geodynamics, Tectonics

## Abstract

The plate tectonic cycle produces chemically distinct mid-ocean ridge basalts and arc volcanics, with the latter enriched in elements such as Ba, Rb, Th, Sr and Pb and depleted in Nb owing to the water-rich flux from the subducted slab. Basalts from back-arc basins, with intermediate compositions, show that such a slab flux can be transported behind the volcanic front of the arc and incorporated into mantle flow. Hence it is puzzling why melts of subduction-modified mantle have rarely been recognized in mid-ocean ridge basalts. Here we report the first mid-ocean ridge basalt samples with distinct arc signatures, akin to back-arc basin basalts, from the Arctic Gakkel Ridge. A new high precision dataset for 576 Gakkel samples suggests a pervasive subduction influence in this region. This influence can also be identified in Atlantic and Indian mid-ocean ridge basalts but is nearly absent in Pacific mid-ocean ridge basalts. Such a hemispheric-scale upper mantle heterogeneity reflects subduction modification of the asthenospheric mantle which is incorporated into mantle flow, and whose geographical distribution is controlled dominantly by a “subduction shield” that has surrounded the Pacific Ocean for 180 Myr. Simple modeling suggests that a slab flux equivalent to ~13% of the output at arcs is incorporated into the convecting upper mantle.

## Abstract (web summary)

We discover a pervasive subduction influence in the Arctic, Atlantic and Indian mantle, which is nearly absent in the Pacific mantle. Such a hemispheric-scale upper mantle heterogeneity reflects the control of a “subduction shield” that has surrounded the Pacific Ocean for 180 Myr.

## Introduction

A classic paradigm of mantle heterogeneity has been that oceanic plates subduct at trenches, a water-rich flux comes off the slab to create arc volcanics, and then mantle heterogeneity is produced by the processing of residual crust and sediment circulating in the mantle^1–3^. The flux off the slab enriches arc volcanics in H_2_O and elements such as Rb, Ba, Th, U, Sr, and Pb relative to the rare earth elements (REE), while the REE themselves are enriched relative to Nb and Ta^4^. These processes have led to a bimodal distribution of certain element ratios, notably Ce/Pb and Nb/U^5^, with ocean ridge (MORB) and ocean island basalts having “canonical” ratios of ~25 and 47^5–9^, and arc volcanics and continent crust having values <10. This division implies that there is a highly efficient filter at subduction zones, where the water-rich flux coming off the slab is restricted to the convergent margin and does not escape to “pollute” the rest of the mantle. Where MORB samples have been found that deviate from these ratios^10–13^, explanations have called upon recycled sediment^12^ or subcontinental lithosphere^13^, or subduction-modified mantle in the Chile Rise^10^ and South Atlantic^11^. In general, incorporation of subduction-modified asthenosphere has been thought to be largely absent from MORB.

**Fig. 1:**
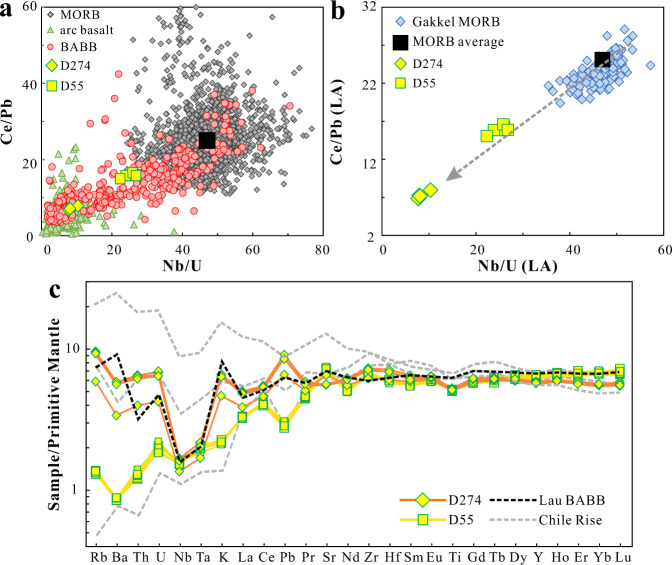
Trace element characteristics of Gakkel mid-ocean ridge basalts. **a** Ce/Pb vs. Nb/U plot for convergent-margin like mid-ocean ridge basalts (MORB) from Gakkel (Dredge 274 & 55). Global MORB and back-arc basin basalts (BABB) from Gale et al.^15^, as well as arc basalts from Turner and Langmuir^63^ are plotted for comparison. **b** New high-quality laser ablation (LA) data of Gakkel MORB show a positive Ce/Pb and Nb/U correlation. Gakkel MORB data were averaged per station (130 stations in total). Arrow shows the trend towards the influence of subduction component in Gakkel MORB. Average Nb/U and Ce/Pb ratios for MORB (black square) are from Hofmann et al.^5^. **c** Primitive mantle normalized trace element diagram for the convergent-margin like Gakkel MORB. Primitive mantle values are from McDonough and Sun^64^. MORB samples from Chile Ridge with convergent margin-like characteristics^10^ and average basalt compositions of Lau back-arc basin from 20–23°S calculated based on Gale et al.^15^ are also plotted.

One tectonic environment with compositions intermediate between MORB and arcs is the back-arc basin spreading center, where back-arc basin basalts (BABB) occur that have higher water contents and intermediate values of Ce/Pb and Nb/U (Fig. 1). BABB are melts of the mantle wedge that has been modified by the hydrous component coming off the slab, which we will refer to as the “slab flux”. They show irrefutably that some of the slab flux escapes from the arc front into the asthenospheric mantle. Might some of this subduction-modified mantle then be incorporated into mantle flow? And where back-arc basins are not present, should not the subduction-modified mantle that would be sampled if there were a back-arc basin contribute to the convecting asthenosphere, where it should occasionally be later sampled by open ocean ridges? To explore these questions requires a careful examination of MORB data to see if a signal akin to BABB might be more widespread than currently realized, and to determine whether this signal is in fact asthenosphere modified by a slab flux, rather than simply dispersed sediment from recycled plates^12^.

Key evidence for this component would reside in element ratios such as Ce/Pb and Nb/U, or ratios of other elements to the universally depleted Nb, all of which are typical characteristics of subduction zone volcanics^4^. The MORB dataset both regionally and globally does in fact contain large variations in Ce/Pb and Nb/U ratios^9,14^ (Supplementary Discussion and Supplementary Fig. 1), and even the filtered global MORB data set of Gale et al.^15^ has Nb/U of 25–75 and Ce/Pb from 10 to >100 (Fig. 1a). Analytical problems for Ce/Pb and Nb/U ratios in MORB historically have been severe, however, resulting from Pb contamination, alteration effects on U, and large uncertainties at low U and Pb concentrations (Supplementary Discussion). The data are clustered around the canonical values that are completely distinct from arc basalts, with BABB having intermediate values (Fig. 1a), but the possibility of recognizing a meaningful signal within the MORB data has been obscured by the various difficulties.

Here, we show a pervasive subduction influence in the Gakkel MORB with high-quality new trace element and water data. Such an influence is also found in Atlantic and Indian MORB but is nearly absent in Pacific MORB, and their geographical distribution is controlled dominantly by a “subduction shield” that has surrounded the Pacific Ocean for 180 Myr.

## Results and discussion

### A subduction signal on the Gakkel Ridge

Laser ablation inductively coupled plasma mass spectrometry (LA-ICP-MS) analyses of glass chips avoid the problems of alteration (U) and contamination (Pb), and permit far larger trace element datasets than can be acquired by laborious ICP-MS solution techniques. To this end, we have developed precise and accurate laser ablation methods, and reanalyzed major and trace elements on the complete set of 576 Gakkel MORB glasses from 130 dredge stations^15–17^ (Supplementary Data 1–3). We unexpectedly discovered arc-like trace element characteristics (Fig. 1) in multiple samples from two dredges, which we confirmed by resampling the dredges to analyze further samples. The most extreme samples have low Nb/U (7–10) and Ce/Pb (6–8), akin to samples from back-arc basins (Fig. 1a), with distinct negative Nb-Ta and positive Pb anomalies (Fig. 1c). These samples also have higher H_2_O/Ce (255–415) than average global MORB (~200^18,19^), higher ^207^Pb/^204^Pb and ^208^Pb/^204^Pb ratios relative to the northern hemisphere reference line, high ^87^Sr/^86^Sr (up to 0.7037) and low ^143^Nd/^144^Nd ratios (~0.5128) (Supplementary Table 1).

**Fig. 2:**
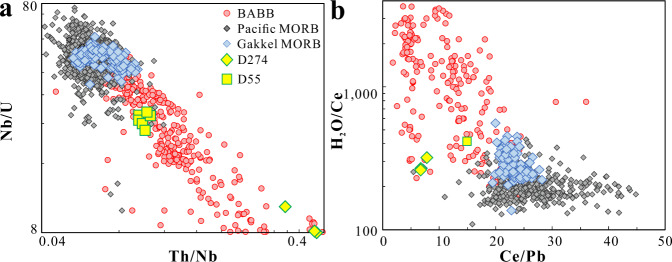
Plots for Nb/U vs. Th/Nb and H_2_O/Ce vs. Ce/Pb for Gakkel and Pacific mid-ocean ridge basalts, as well as back-arc basin basalts. Gakkel samples shift to higher Th/Nb, and H_2_O/Ce, as well as lower Nb/U, and Ce/Pb ratios, indicating a pervasive subduction influence in the Arctic mantle. Station averages were plotted for Gakkel samples as in Fig. 1b.

These data raise the question whether there might be a more pervasive subduction influence in this region. Indeed, the positive correlation between Nb/U and Ce/Pb ratios from the new laser data suggests a subduction influence on the Gakkel MORB (Fig. 1b), which is observed in elevated Th/Nb and H_2_O/Ce as well (Fig. 2). In all, 154 new water measurements (Supplementary Data 2) conducted in this study suggest that the Gakkel MORB have a significant water enrichment compared to other MORB globally, with an average H_2_O/Ce of 289 ± 61, compared to 205 ± 49 for Pacific MORB (average from our data compilation). High H_2_O/Ce and Th/Nb ratios coupled with low Nb/U and Ce/Pb ratios support subduction contributions to the Gakkel mantle (Fig. 2 and Supplementary Discussion).

**Fig. 3:**
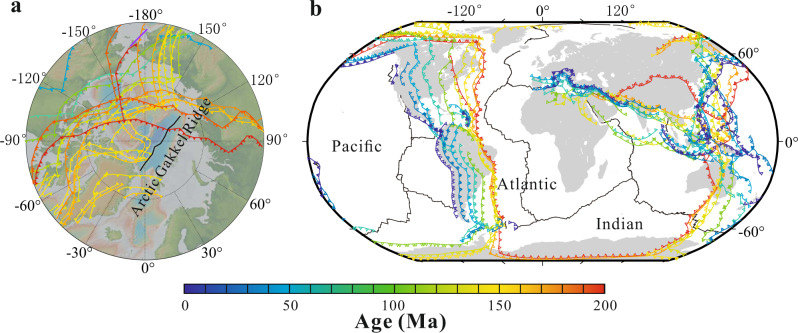
Subdution zone locations during the past 200 Myr. Reconstructed subduction locations during the past 200 Myr for **a** Arctic Ocean, and **b** Indian and Atlantic Oceans, modified from Shephard et al.^21^ and Young et al.^65^, respectively.

Although no proximal subduction zone is present to provide slab-derived materials, the Arctic has been surrounded by subduction zones during the past 200 Myr, for example, Mogol-Okhotsk Plate from >200–150 Ma^20^, Farallon Plate from 200 to 100 Ma^20^, South Anuyi Plate from ~120 to 160 Ma^21^ and Izanagi Plate from >200 to 50–55 Ma^20,21^ (Fig. 3a and Supplementary Fig. 4). It appears a portion of the water-rich slab flux was incorporated in the mantle wedge corner flow and escaped the arc. Some of this subduction-modified mantle could then contribute to the asthenosphere that was later sampled when the Gakkel ridge began to spread. Plate reconstruction^21^ suggests that these past subduction zones, and their associated metasomatized sub-arc mantle, were located beneath or near the current spreading Gakkel ridge (Fig. 3a). Such a scenario would provide a slab flux to the Arctic mantle to contribute to the current MOR magmatism.

### Identifying subduction signals in global mid-ocean ridge basalts

The observation of a subduction influence on the Gakkel Ridge leads to expectations of a more widespread effect. Plates in the Pacific Ocean have had long subduction histories beneath the Eurasian and American plates as well as the Arctic^20,21^ (Fig. 3), leading to the possibility that subduction signals could be present in the Indian and Atlantic Oceans as well. In contrast, the blanket of subduction zones around the Pacific would shield the Pacific basin from subduction-modified mantle wedges, leading to the expectation of little subduction signal for Pacific ridges.

We update here the global data base of Gale et al.^15^, with recently published high-quality data (Supplementary Data 4). The final data set adds ~3000 new analyses to the Gale compilation (not including the new Gakkel data) that can be used to evaluate the subduction signal on a global basis.

**Fig. 4:**
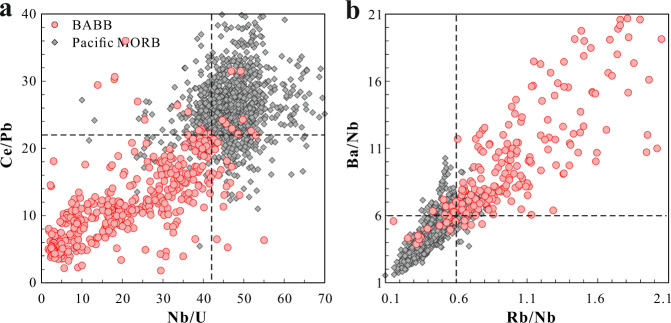
Trace element systematics of global back-arc basin basalts. Plots of **a** Ce/Pb vs. Nb/U and **b** Ba/Nb vs. Rb/Nb for global back-arc basin basalts, as well as Pacific mid-ocean ridge basalts. Note the clean separation of the two sets of data. Global back-arc basin basalts, including volcanics from the Lau, Mariana Trough, Scotia and Manus back-arc basins, can be well distinguished with Ba/Nb and Rb/Nb ratios higher than 6 and 0.6, and Ce/Pb and Nb/U ratios lower than 22 and 42 (shown by the dashed lines), respectively. Data sources can be found in the Supplementary Data 4.

The appropriate comparison for the signal that escapes the arc is not the arc front itself, which reflects the captured slab flux, but the BABB that reflect a subduction signal that emerges beyond the arc at a spreading ridge. To cope with potential problems with literature Pb and U data, additional ratios sensitive to subduction such as Ba/Nb and Rb/Nb are needed. Over 94% of global BABB have Ba/Nb > 6 and Rb/Nb > 0.6, and over 92% of them have Nb/U < 42 and Ce/Pb < 22 (Fig. 4). Therefore, these four ratios can be used to construct a “BABB filter” such that if at least three of the four ratios meet the BABB filter, the sample has a subduction influence. Using any 3 instead of all four leaves some room for analytical error, in case a real BABB-like MORB has a bad Pb or U measurement. This filter provides a simple true or false test for subduction influence (H_2_O/Ce ratios are not included in the filter because of the rather limited literature data on H_2_O, but are discussed further below). The efficacy of the BABB filter is good: 94% of global BABB data, yet only 2% of Pacific MORB (apart from the southern Chile Ridge) pass the filter. For the Gakkel Ridge, 229 of 710 samples pass the filter, showing a pervasive subduction influence.

For the global ocean ridge system, of the 4353 samples with at least three of the ratios available to apply the BABB filter, 550 samples pass the filter, leading to the conclusion that 13 percent of global MORB, and over 17% of MORB outside the Pacific basin, contain an observable subduction influence. We refer to these samples as “BABB-like MORB.”

**Fig. 5:**
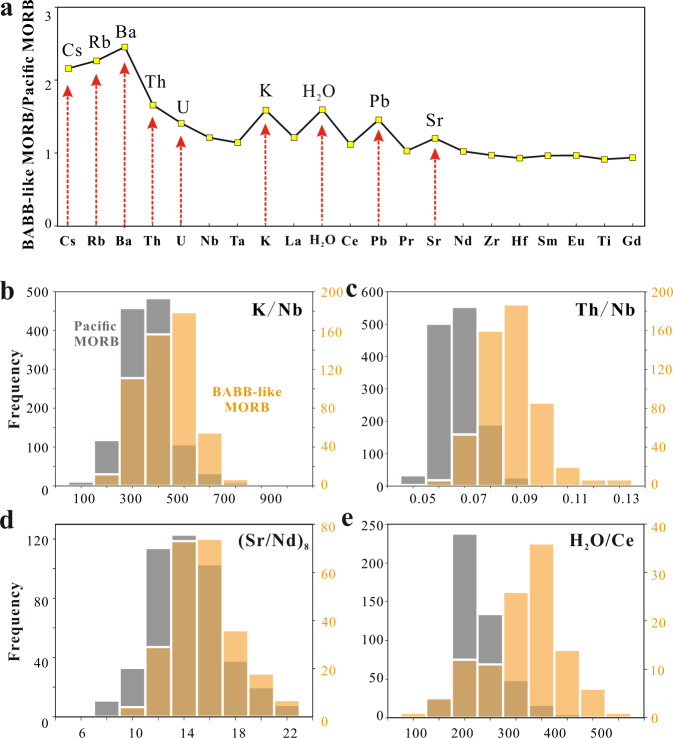
Chemical variations of mid-ocean ridge basalts with back-arc signatures relative to Pacific mid-ocean ridge basalts. **a** Enrichment factors of average mid-ocean ridge basalts with back-arc signatures (BABB-like MORB) relative to average Pacific MORB. BABB-like MORB are enriched in Cs, Rb, Ba, Th, U, K, H_2_O, Pb, and Sr relative to Pacific MORB (shown by the red dashed arrows). Log-normal mean values were calculated as the averages for both groups to approximate the median value of each dataset and are less influenced by the few high concentrations^15^. The average Pacific MORB compositions were calculated using Pacific MORB with MgO >4 wt.%, so that the averages of the two groups have comparable MgO to minimize the effect of variable magma differentiation on the calculated results. **b**–**e** Histograms of K/Nb, Th/Nb, (Sr/Nd)_8_, and H_2_O/Ce for Pacific MORB and BABB-like MORB. BABB-like MORB are significantly different in all these ratios relative to Pacific MORB determined by Student’s *t* test (*P* < 0.001). (Sr/Nd)_8_ ratios are the Sr/Nd ratios of MORB with MgO between 7.5 and 8.5 wt.% to exclude the effect of varied degree of plagioclase crystallization on Sr variations. Darker brown shades are where the separate distributions for Pacific MORB (gray columns) and BABB-like MORB (brown columns corresponding to the *y*-axis to the right of each figure) overlap.

Compared with Pacific MORB, trace element distributions in BABB-like MORB show enrichments not only in Rb, Ba, U, and Pb (elements included in the BABB filter), but also show clear peaks in Cs, K Th, and H_2_O, as well as a slight enrichment in Sr (Fig. 5a). Global BABB-like MORB have higher H_2_O/Ce ratios of 293 ± 75 compared to those from Pacific MORB (205 ± 49), consistent with a slightly hydrated mantle source with BABB signatures. Histograms of K/Nb, Th/Nb, and Sr/Nd all show statistically significant increases in BABB-like MORB compared to Pacific MORB, lending support to the validity of the filter (Fig. 5b–e). All these elements and ratios are enriched in arc volcanics relative to Pacific MORB, consistent with BABB-like MORB being caused by a water-rich slab flux.

**Fig. 6:**
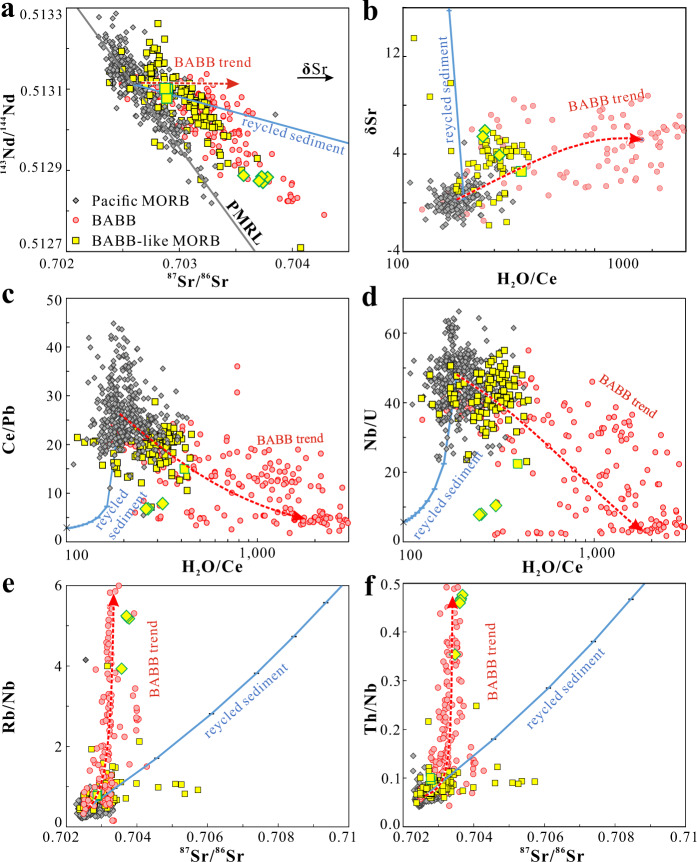
Chemical characteristics of mid-ocean ridge basalts with back-arc signatures. **a** ^87^Sr/^86^Sr vs. ^143^Nd/^144^Nd, and **b** δSr (deviation of ^87^Sr/^86^Sr relative to the Pacific mid-ocean ridge basalts (MORB) reference line (PMRL), details of this line can be found in the Methods section) vs. H_2_O/Ce ratios, **c**, **d** H_**2**_O/Ce vs. Ce/Pb, and Nb/U, **e**, **f** ^87^Sr/^86^Sr vs. Rb/Nb, Th/Nb, and for Pacific MORB, back-arc basin basalts (BABB), and BABB-like MORB. The BABB-like MORB have higher H_2_O/Ce, and Th/Nb and shift to higher ^87^Sr/^86^Sr relative to ^143^Nd/^144^Nd ratios than Pacific MORB. Red curves show the BABB trends, indicating the direction for subduction input. Blue curves show the trends for mixing recycled sediments in Pacific MORB mantle with 1% increments. Pacific MORB mantle compositions were calculated assuming the average Pacific MORB are formed by ~10% melting of their source mantle, and the global subducting sediment compositions^30^ were used as the recycled sediment endmember with a H_2_O/Ce ratio of 100 (details on the H_2_O/Ce estimation can be found in the Methods section). Recycled sediment fails to account for the characteristics of most BABB-like MORB. Data source can be found in the Supplementary Data 4.

Further support for the BABB influence comes from a comparison of the Sr–Nd isotopes. Sr is mobilized preferentially relative to Nd coming off the slab^22^, leading to higher ^87^Sr/^86^Sr ratios for the same ^143^Nd/^144^Nd values in arc basalts. This offset is also present in BABB themselves. BABB-like MORB also show the offset to higher ^87^Sr/^86^Sr relative to Pacific MORB (Fig. 6a, b), and such an offset (**δ**Sr, defined as the deviation of ^87^Sr/^86^Sr relative to the Pacific MORB reference line (PMRL), details can be found in the Methods section) generally correlated with higher H_2_O/Ce ratios for BABB-like MORB compared to Pacific MORB (Fig. 6b).

An alternative hypothesis for these observations is that they are caused by mixing of recycled sediments^12^ or continental crust into the mantle source^2^. It would then not be the slab flux, but the slab itself that would mix with the asthenosphere to cause the heterogeneity. This model, for example, has been widely used to explain mantle heterogeneities observed in ocean island basalts^1,23–26^ as well as MORB^12,27^. The geodynamic implications of these two models differ greatly. One hypothesis calls upon incorporating a portion of the water-rich flux off the slab into the convecting upper mantle asthenosphere. The second would call on deep subduction and mixing of dehydrated sediments. Both processes could lead to samples passing the “BABB filter”, since dehydrated sediments would presumably still have low Ce/Pb and Nb/U and high Rb/Nb and Ba/Nb ratios, but the term BABB filter would obviously no longer be appropriate.

Three sets of evidence support a water-rich slab flux as the main cause for the chemical variations of BABB-like MORB. First, samples that pass the BABB filter mostly have high H_2_O/Ce ratios relative to Pacific MORB, whereas recycled sediments are dry with H_2_O/Ce of lower than 100 due to efficient dehydration beneath arcs^28^. Continental crust is dry as well, with H_2_O/Ce of ~120 for upper crust and ~50 for the lower crust (calculation on H_2_O/Ce of continental crust can be found in the Methods section). Second, arc volcanics and BABB have much higher Sr/Nd ratios than MORB mantle, owing to higher mobility for Sr relative to Nd in the water-rich slab flux^22^. BABB-like MORB reflect this feature of the slab flux with higher (Sr/Nd)_8_ (~15) than Pacific MORB (~13, Fig. 5d), higher than the Sr/Nd ratios of sediments/upper continental crust^29,30^ (~11). Third, the slab flux has high ratios of many slab-derived elements to Nb, but only slightly elevated ^87^Sr/^86^Sr ratios (<0.704). Continental crust has similarly high trace element ratios, but significantly elevated ^87^Sr/^86^Sr (e.g. ^87^Sr/^86^Sr = 0.7124^30^). Mixing enough sediment to double ratios such as Ba/Nb and Rb/Nb would lead to ^87^Sr/^86^Sr > 0.704, which is not observed for most BABB-like MORB (Fig. 6). While a handful of samples that pass the filter indeed have anomalously high ^87^Sr/^86^Sr and low H_2_O/Ce which might call for alternative explanation, the majority of BABB-like MORB have coupled moderate enrichments in ^87^Sr/^86^Sr and H_2_O/Ce, which are mostly intermediate between Pacific MORB and BABB (Fig. 6). All these observations suggest that the water-rich slab flux is mainly responsible for the occurrence of most BABB-like MORB.

### Quantitative assessment of the slab flux in the source of global mid-ocean ridge basalts

To quantify the relative contributions of the slab flux to global MORB mantle, we assume Nb in the source of BABB-like MORB receives minimal contribution from the subducted slab, and the slab flux can be calculated by the relative enrichment of elements (e.g., Cs, Rb, Ba Th, U, and K) which are known to be contributed by slab-derived fluids/melts^31^ (Fig. 5a) over Nb. The ambient mantle (AM) without subduction influence is assumed to have the Cs/Nb, Rb/Nb, Ba/Nb, U/Nb, Th/Nb, and K/Nb ratios of average Pacific MORB, and these ratios (e.g. (Rb/Nb)_AM_) would not change greatly during moderate degrees of mantle melting for MORB and arc magma genesis as they are all highly incompatible elements. Therefore, the slab flux (***f***) for each element in BABB-like MORB (***f*** ^B-MORB^), Rb, for example, can be estimated by the average Rb concentrations in BABB-like MORB multiplied by the total melt volume of BABB-like MORB (*V*_B-MORB_), minus the ambient mantle Rb contributions before subduction input which can be estimated by Nb concentrations and Rb/Nb ratios of ambient mantle.

1 $${{{{{{\boldsymbol{f}}}}}}}_{{{{{{\rm{Rb}}}}}}}^{{{{{{\rm{B}}}}}}-{{{{{\rm{MORB}}}}}}}=({{{{{\rm{Rb}}}}}}_{{{{{{\rm{B}}}}}}-{{{{{\rm{MORB}}}}}}}-{{{{{{\rm{Nb}}}}}}}_{{{{{{\rm{B}}}}}}-{{{{{\rm{MORB}}}}}}}{({{{{{\rm{Rb}}}}}}/{{{{{\rm{Nb}}}}}})}_{{{{{{\rm{AM}}}}}}})\times {V}_{{{{{{\rm{B}}}}}}-{{{{{\rm{MORB}}}}}}}$$

This can then be compared to the slab flux released beneath arc and BABB:

2 $${{{{{{\boldsymbol{f}}}}}}}_{{{{{{\rm{Rb}}}}}}}^{{{{{{\rm{arc}}}}}}}=({{{{{\rm{Rb}}}}}}_{{{{{{\rm{arc}}}}}}}-{{{{{{\rm{Nb}}}}}}}_{{{{{{\rm{arc}}}}}}}{({{{{{\rm{Rb}}}}}}/{{{{{\rm{Nb}}}}}})}_{{{{{{\rm{AM}}}}}}})\times {V}_{{{{{{\rm{arc}}}}}}}$$

3 $${{{{{{\boldsymbol{f}}}}}}}_{{{{{{\rm{Rb}}}}}}}^{{{{{{\rm{BABB}}}}}}}=({{{{{\rm{Rb}}}}}}_{{{{{{\rm{BABB}}}}}}}-{{{{{{\rm{Nb}}}}}}}_{{{{{{\rm{BABB}}}}}}}{({{{{{\rm{Rb}}}}}}/{{{{{\rm{Nb}}}}}})}_{{{{{{\rm{AM}}}}}}})\times {V}_{{{{{{\rm{BABB}}}}}}}$$

Melt production at global ocean ridges has been estimated to be 21 km^3^/year^32^, and the melt volume of BABB-like MORB which make up 13% of global ocean ridge production, is ~2.73 km^3^/year. The melt volume at global arcs is estimated to be ~2 km^3^/year, calculated with the average arc magmatic addition rate of 40 km^3^/Ma/km^33,34^, along with the ~50,000 km length of global arcs^35^. The melt volume of global BABB can be estimated to be ~1.3 km^3^/year using the spreading rate and segment length in Gale et al.^15^, and an average oceanic crustal thickness of 6 km. Average compositions of Pacific MORB, BABB-like MORB, BABB, and arc volcanics can be found in Supplementary Table 2.

These equations can then be used to estimate the slab flux of Cs, Ba, U, Th, and K erupted in BABB-like MORB, BABB, and arc volcanics, to arrive at an average estimate of the slab flux that escapes the convergent margin to be incorporated into upper mantle flow. What proportion of the slab flux would need to make it into the convecting mantle to produce the signal observed in MORB? The portion of slab flux beneath MORB compared to arc can be calculated by ***f***^B-MORB^/***f***^arc^ for each element. The result shows that a flux equivalent to ~13% of the erupted flux at convergent margins, and slightly lower than the flux coming out at back-arc basins, escapes beyond the subduction zone into the convecting mantle, later to be incorporated into ocean ridge volcanism. (Table 1).

**Table 1:** Estimated slab fluxes for Cs, Rb, Ba, U, Th, and K in MORB and BABB relative to the global output at arcs.

	Cs	Rb	Ba	U	Th	K	Average
***f***^B-MORB^/***f***^arc^ (%)	6	22	14	6	17	12	13
***f***^BABB^/***f***^arc^ (%)	16	27	15	17	18	14	18

Slab flux averaged for all the elements are listed in the last column.

A possible caveat is that global BABB-like MORB tend to occur on slower spreading ridges outside the Pacific ocean basin, which means that the melt volume of BABB-like MORB can be lower than the estimated 2.73 km^3^/year, and our result can be considered as a maximum estimate of the slab flux beneath global MORB mantle. It is important to note that current back-arc basins show a substantial range in compositions, and are all behind oceanic island arcs. The BABB-like MORB, on the other hand, mostly occur behind ocean-continent subduction zones. Such a variation in subduction modes may lead to varied slab flux compositions, as multiple mechanisms may generate the slab flux released beneath arcs^36,37^. That said, all forms of slab flux should have the characteristic element ratios used in the current evaluation.

Based on the widely held idea that Nb/U and Ce/Pb of oceanic basalts show only small deviations from the “canonical values”, it has seemed that the slab fluxes manifest at volcanic arcs and back-arcs rarely appear at ridges, or that the influence has been globally uniform^6^. The new data presented here suggest instead that a small but significant fraction of the slab flux can be widely and heterogeneously disseminated in the shallow asthenospheric mantle as a contributing factor to mantle heterogeneity sampled at ocean ridges.

### Geographical pattern of the subduction influence and the Pacific subduction shield

**Fig. 7:**
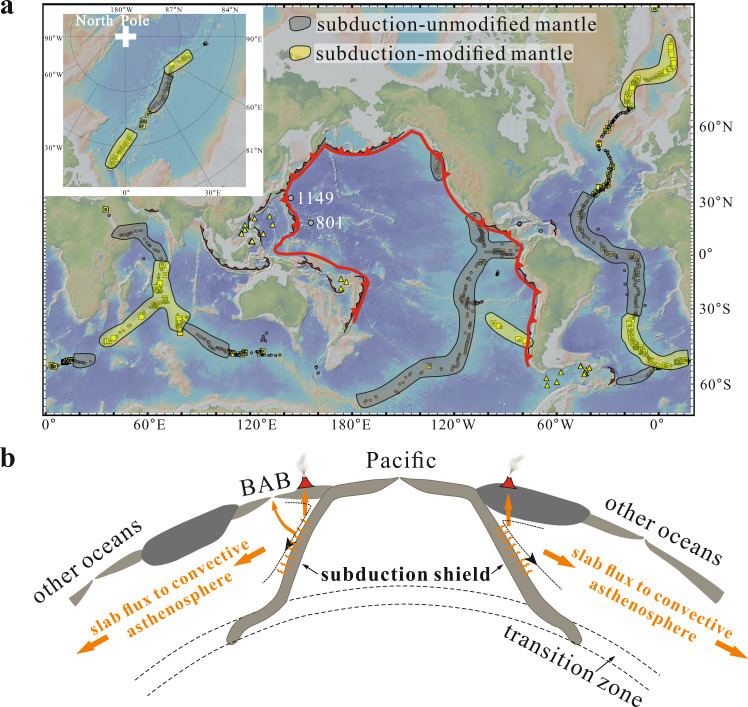
The subduction influence on ocean ridge basalts outside the Pacific subduction shield. **a** Distribution of global mid-ocean ridge basalts with back-arc signatures (yellow squares) and **b** schematic model of the subduction shield. **a** Mid-ocean ridge basalts (MORB) samples which do not pass the back-arc basin basalt (BABB) filter (gray dots) are also plotted. The majority of the global BABB-like MORB (yellow symbols) are distributed outside the Pacific subduction zones shown by the red lines. Yellow-shaded blobs denote ridge sections where >20% of MORB pass the BABB filter, indicating the presence of subduction-modified mantle beneath the ridges. Gray-shaded sections denote sections where <3% of MORB pass the BABB filter, suggesting the dominance of subduction-unmodified mantle beneath the ridges. The circum-Pacific subduction zones are shown by the thick red curves. **b** The circum-Pacific subduction zones form a subduction shield to prevent subduction contribution to Pacific MOR magmatism. Slab flux (water-rich component off the slab, represented by orange curves) would metasomatize the overlying mantle wedge right above the subducted slab. The metasomatized mantle would partially be dragged downwards by corner flow at mantle wedge and would not contribute to the arc volcanism. They would eventually mix into the convecting mantle to feed BAB and MOR magmatism. Old Pacific MORB glasses from drilled holes 1149 and 801^40^ (light gray dots) all have none ratios passing the BABB filter, and basalts from the Fiji^66^, Sulu Sea^67^, South China Sea^68^, Philippine Sea Plate^31,69^, and Drake Passage^70,71^ (yellow triangles) outside the Pacific subduction zones have BABB signatures, consistent with the subduction shield model. Global MORB data source can be found in the Supplementary Data 4. Bathymetry from GeoMapApp^72^.

The subduction influence we have identified has clearly defined regional variations. The subduction influence is present in only 2% of Pacific MORB, 10% for the Atlantic as a whole, 24% for the Indian, and 32% for Gakkel MORB. Along the MAR, there are also clear provinces. A large expanse of ocean ridge in the equatorial Atlantic between 33°N and 34°S has <3% of samples passing the BABB filter, similar to the Pacific. Where the Chile Ridge is being subducted beneath South America, opening a slab window into the arc-influenced mantle wedge^38^, there is a clear BABB signal. These spatial regularities can then be mapped by delineating ridges that have <3% and those that have >20% BABB-like MORB (Fig. 7a). The former is Pacific-like with minimal subduction input, and the latter receives considerable slab flux.

What is striking is the lack of subduction influence apart from the Chile Ridge in the Pacific Ocean. If the cause of the observations presented here is indeed subduction, the lack of subduction influence in the Pacific can be understood from global tectonic reconstructions that show that around the Pacific Ocean there has been a continuous “subduction shield” (Fig. 7b) for at least the last 180 million years^20^. Tomographic studies suggest circum-Pacific subducted slabs are mostly continuous at least to transition zone depth^39^ (Fig. 7b), and therefore, this subduction shield would strictly limit the contribution of the slab flux to sub-Pacific mantle, simply explaining the striking absence of this component in Pacific MORB today. In this case, older Pacific basalts should also have no subduction influence, which is supported by the fact that no MORB glasses from the oldest Pacific crust drilled at Holes 801 and 1149^40^ pass the BABB filter (Fig. 7a). In contrast, in older samples outside the subduction shield in the western Pacific, BABB-influenced samples are pervasive (Fig. 7a). These observations are consistent with the continuous releasing of slab flux outside the Pacific subduction zones. Therefore, we propose that the water-rich component off the subducted slab, termed slab flux in this study (Fig. 7b), would metasomatize the overlying mantle wedge right above the subducted slab. Some of the metasomatized mantle would partially be dragged downwards by corner flow at mantle wedge^41,42^ away from arc, mixing into the convecting mantle and contributing to BAB and MOR magmatism. Some slab flux might also metasomatize sub-lithospheric mantle, which could later be incorporated into mantle flow to be sampled at ocean ridges.

Even outside the Pacific basin there are important regional differences. The equatorial Atlantic between 33°N and 34°S, as well as the easternmost Indian Ocean have Pacific-like signatures, with less than 3% of samples passing the BABB filter (Fig. 7a). Such an observation provides additional evidence for the outflow of Pacific mantle to Atlantic and Indian as a consequence of shrinking Pacific Ocean^43^. The initially proposed gateways for Pacific mantle outflow were the Caribbean, Southeast Indian Ocean and Drake Passage^43^. Our study confirms that the Caribbean and easternmost Southeast Indian Ocean with Pacific-like trace element signatures are likely gateways for Pacific mantle outflow (Fig. 7a), whereas the Drake passage, with strong subduction signals (Fig. 7a), might not be such a gateway. Such an inference is consistent with previous study based on Pb isotopes^44–47^ and geophysical studies^48–52^.

An obvious question is the relationship between the signal we have identifed and the “Dupal” isotopic anomaly, which was identified in the Atlantic and Indian Oceans with elevated ^208^Pb/^204^Pb (and ^207^Pb/^204^Pb) for given ^206^Pb/^204^Pb ratios^16,53,54^. There is spatial coherence between the two anomalies, partiuclaly in the South Atlanic and western Indian oceans. The influence we have identified, however, is distinct from Dupal. Sixty-one percent of global MORB samples with a Dupal isotopic signature of △8/4 (elevated ^208^Pb/^204^Pb to ^206^Pb/^204^Pb) >30^16,54^ do not pass the BABB filter. The eastern part of the Southeast Indian Ridge, for example, has a strong Dupal signature but no BABB-like MORB samples (Fig. 7a and Supplementary Figs. 5–6). Furthermore, MORB with extreme Dupal signature (△8/4~100) are dry with Pacific-like H_2_O/Ce ratios (<200), thus are not likely to be accounted for by recent subduction contributions. Instead, the recent slab flux input was superimposed on whatever background mantle it mixed with. It is intriguing that the Dupal anomaly as well lies outside the Pacific subduction shield, which may be traced back to 400 Ma when the Paleo-pacific Ocean started to subduct beneath the Gondwana supercontinent^55^.

Subduction is a global process and the subduction influence on the back-arc basins beyond the volcanic fronts of convergent margins has long been known. This influence must reside in the upper mantle, and upon reflection, it has been mysterious that this influence has been so rarely described in ocean ridge basalts. The observations presented here demonstrate that there is indeed a widespread subduction influence on MORB caused by a slab flux into the convecting upper mantle, manifested clearly in 17% of global MORB samples outside of the Pacific Ocean basin. The geographical distribution of this influence can be understood in the context of the long-term tectonic history of plate movements and the global distribution of subduction zones.

## Methods

### Trace elements

The major and trace elements were measured using a RESOlution-S155 193 nm Excimer laser connected to a Thermo iCAP Q ICP-MS which is used only for laser ablation analyses at Harvard University. Two points per chip were obtained, and the values reported are the average of the two analyses. Ca contents of the major element analysis of the same chip were used as an internal standard. Data were then reduced using basalt standard BHVO-2G run in the same run (values in Supplementary Data 1), which was also used as a drift correction standard run every 10 analyses. The Harvard lab basalt glass standard VE32 was present in every probe mount, and was analyzed as an unknown with the other samples to get estimates of between run reproducibility over the course of this study. VE32 data were very consistent between runs (Supplementary Data 1), and provide good estimates of the errors in the analyses, which generally scale with concentration (Supplementary Data 1).

### Water

Concentrations of H_2_O dissolved in glasses were analyzed by Fourier Transform Infrared Spectroscopy (FTIR) at the University of Tulsa using published methods and calibrations^56,57^ with slight modifications^18^. Doubly polished glass wafers, 100–250 μm thick, were placed atop a 2-mm-thick KBr pellet and analyzed using a NicPlan IR microscope equipped with a HgCdTe detector, attached to a Nicolet 520 FTIR. Thickness was measured by two methods: firstly by digital micrometer, and secondly by focusing the calibrated z-axis of the FTIR microscope stage on the glass wafer and on the adjacent KBr disk using reflected light. Optically clear areas of known thickness (±2 μm), 80 × 80 μm, were analyzed with 256 scans/spot. Absorbance at the broad 3550 cm^−1^ (combined OH and H_2_O) and 1630 cm^−1^ (molecular H_2_O only) peaks were measured after subtraction of interpolated backgrounds. Density was assumed to be 2.8 g/cm^3^. Molar absorption coefficients used for all glasses were: 63 l/mol-cm for 3550 and 25 l/mol-cm for e1630. Analyses are the average of 3–4 spot determinations of 3550 cm^−1^ on two separate wafers. Replicate analyses of different wafers from the same specimen were typically reproducible to ±5%.

### Sr-Nd-Pb isotopes

Column chromatographic procedures for Sr-Nd-Pb purifications were carried out in Class 100 or Class 1000 ultra-clean chemistry lab at Lamont-Doherty Earth Observatory following routine procedures^58^. Hand-selected clean glass chips were sonicated in cold 8 N HNO_3_ in the sonicator for 10 min and washed with quartz-distilled water 4 times prior to standard HNO_3_-HF hotplate digestions. Pb was extracted using BioRad® AG1-X8 resin. Sr was extracted using Eichrom Sr resin. Rare Earth Elements (REE) were extracted using Tru-Spec® resin. Nd was extracted from the REE cut either using alpha-hydroxyisobutyric acid (alpha-HIBA) and BioRad® AG50-X8 resin or using Eichrom® Ln resin and 0.22 N HNO_3_ acid.

Isotopes were measured on a VG-Axiom multicollector-inductively coupled mass spectrometer (MC-ICPMS). In-run mass fractionations are normalized using ^86^Sr/^88^Sr = 0.1194, and ^146^Nd/^144^Nd = 0.7219. All samples were further normalized to the ^86^Sr/^88^Sr value of 0.71024 for SRM 987, ^143^Nd/^144^Nd value of 0.511858 for La Jolla, and ^206^Pb/^204^Pb, ^207^Pb/^204^Pb, and ^208^Pb/^204^Pb values of 16.9356, 15.4891, and 36.7006 for NBS 981^59^, respectively.

Pb total procedure blank is always below 100 pg. As procedural blanks are negligible given the sample size and current analytical uncertainties, no blank corrections were performed. Measurements of international standard sample BCR-2 (after acid leach) yielded 0.704998 ± 0.000013 (2σ, *n* = 6) for ^87^Sr/^86^Sr, 0.512609 ± 0.000006 (2σ, *n* = 3) for ^143^Nd/^144^Nd, as well as 18.7895 ± 0.0008, 15.6523 ± 0.0007, and 38.8845 ± 0.0019 (2σ, *n* = 1) for ^206^Pb/^204^Pb, ^207^Pb/^204^Pb, ^208^Pb/^204^Pb ratios, respectively, which is consistent with the published values of Weis et al.^60^: ^87^Sr/^86^Sr = 0.705019 ± 0.000016 (2σ, *n* = 13), ^143^Nd/^144^Nd = 0.512634 ± 0.000012 (2σ, *n* = 11), ^206^Pb/^204^Pb = 18.8000 ± 0.0020, ^207^Pb/^204^Pb = 15.6236 ± 0.0016, and ^208^Pb/^204^Pb = 38.8244 ± 0.0034 (2σ, *n* = 2).

### The advantage of using Rb/Nb, Ba/Nb, Nb/U, and Ce/Pb to construct the BABB filter

Rb, Ba, Nb, and U as well as Ce and Pb are known to have similar incompatibility during magmatic processes, respectively, thus these element pairs would not be significantly fractionated from each other and these ratios should reflect source characteristics. There are, however, complicating factors. Alteration increases Rb and U contents^40^ relative to Nb, contamination increases Pb relative to Ce, and mantle metasomatism by very low degree melt would increase Rb and Ba over Nb. Thus, various geological processes would lead to variations in each of the four ratios that would not be caused by subduction influence. Using any three of these four ratios to pass the filter minimizes the possibility of alteration, contamination and source enrichment effect leading to a spurious conclusion with respect subduction influence, and also means that a single anomalous ratio does not rule out that influence. Also, in case a real BABB-like MORB has a bad Pb or U measurement, using any three instead of all four leaves some room for analytical error.

### Estimation of H_2_O/Ce ratios for continental crust and recycled sediment

The upper continental crust can contain up to 8000 ppm water, mainly in hydrous minerals or as fluid inclusions in felsic minerals such as quartz and feldspars^61^ and lower continental crust is estimated to contain water of <1000 ppm according to studies of granulite xenoliths^62^. The H_2_O/Ce ratios, which could then be calculated with average Ce contents of continental crust^29^, are 127 for upper continental crust, and 50 for lower continental crust. The recycled subducted sediments are also dry, estimated to have H_2_O/Ce ratios lower than 100^28^. Therefore, contribution of continental crust materials/recycled sediments would result in lower H_2_O/Ce ratios, contrary to the subduction contributions.

### Calculation on Sr isotopic deviation relative to Nd isotopes (δSr)

Pacific MORB can be used to set up a reference line (PMRL) to calculate the deviation of ^87^Sr/^86^Sr relative to ^143^Nd/^144^Nd ratios: **δ**Sr.

4 $$\delta {{{{{\rm{Sr}}}}}}=({\,\!}^{87}{{{{{\rm{S}}}}}}{{{{{\rm{r}}}}}}/{\!\,}^{86}{{{{{\rm{S}}}}}}{{{{{\rm{r}}}}}}-2.0492+2.6244\times {\,\!}^{143}{{{{{\rm{N}}}}}}{{{{{\rm{d}}}}}}/{\!\,}^{144}{{{{{\rm{N}}}}}}{{{{{\rm{d}}}}}})\times {10}^{4}$$

## Supplementary information

Supplementary Information

Description of Additional Supplementary Files

Dataset 1

Dataset 2

Dataset 3

Dataset 4

## Data Availability

All data supporting the findings of this study are available within the paper and supplementary information and data files (Supplementary Tables 1 and 2 and Data Files 1–6).
