# Author Correction: A subduction influence on ocean ridge basalts outside the Pacific subduction shield

**DOI:** 10.1038/s41467-021-26378-6

**Published:** 2021-10-11

**Authors:** A. Y. Yang, C. H. Langmuir, Y. Cai, P. Michael, S. L. Goldstein, Z. Chen

**Affiliations:** 1 State Key Laboratory of Isotope Geochemistry, Guangzhou Institute of Geochemistry, Chinese Academy of Sciences, Guangzhou, China; 2 Key Laboratory of Ocean and Marginal Sea Geology, Guangzhou Institute of Geochemistry, Chinese Academy of Sciences, Guangzhou, China; 3 CAS Center for Excellence in Deep Earth Science, Guangzhou, China; 4 Department of Earth and Planetary Sciences, Harvard University, Cambridge, MA USA; 5 Lamont-Doherty Earth Observatory, Columbia University, New York, NY USA; 6 University of Tulsa, Tulsa, OK USA

**Keywords:** Geochemistry, Geodynamics, Tectonics

Correction to: *Nature Communications* 10.1038/s41467-021-25027-2, published online 06 August 2021.

The original version of this Article contained an error in the author list affiliations, which was previously given as Charles Langmuir is from ‘State Key Laboratory of Isotope Geochemistry, Guangzhou Institute of Geochemistry, Chines Academy of Sciences, Guangzhou, China’ and the ‘Department of Earth and Planetary Sciences, Harvard University, Cambridge, MA, USA’. The correct version states that Charles Langmuir is from the ‘Department of Earth and Planetary Sciences, Harvard University, Cambridge, MA, USA’. This has been corrected in both the PDF and HTML versions of the Article.

