# Supplementary material for: A subduction influence on ocean ridge basalts outside the Pacific subduction shield
Source: Nat Commun. 2021 Aug 6;12:4757. doi: 10.1038/s41467-021-25027-2 (PMC8346520; doi:10.1038/s41467-021-25027-2)
Supplement: Supplementary file 1 — Supplementary Information [file 41467_2021_25027_MOESM1_ESM.pdf]

A subduction influence on ocean ridge basalts outside the Pacific subduction shield

A. Y. Yang[1,2,3], C. H. Langmuir[4,1], Y. Cai[5], P. Michael[6], S. L. Goldstein[5], Z. Chen[4]

[1]State Key Laboratory of Isotope Geochemistry, Guangzhou Institute of Geochemistry, Chinese Academy of Sciences, Guangzhou, China, [2]CAS Center for Excellence in Deep Earth Science, Guangzhou, China. [3]Southern Marine Science and Engineering Guangdong Laboratory (Guangzhou), Guangzhou, China, [4]Department of Earth and Planetary Sciences, Harvard University, MA, USA. [5]Lamont-Doherty Earth Observatory, Columbia University, NY, USA. [6]University of Tulsa, OK, USA

Supplementary Discussion

1. Analytical problems with U and Pb and the compilation of an updated global MORB dataset

The advent of Inductively Coupled Plasma Mass Spectrometry (ICP-MS) analyses has led to large numbers of samples with measurements of Pb and U in ocean ridge basalts. Although MORB have been known to have the "canonical ratios" of Nb/U =47, and Ce/Pb = 25[1,2], yet a download of ICP-MS data on supposedly pristine glasses from any region will show large variations in these ratios. Supplementary Fig S1 a-c shows ICP-MS data from three regions, not chosen for particularly large variability- the Pacific-Antarctic Ridge (PAR, all glasses) (Supplementary Fig. 1a), Mid-Atlantic 33-41°N with glass and whole rock with separate symbols (Supplementary Fig S1b), Central Indian Ridge (all glasses, Supplementary Fig S1c), and the Gakkel Ridge (all glasses, Supplementary Fig. 1d). The few Central Indian Ridge samples with low Nb/U and Ce/Pb appear to be "arc-like", but they do not show negative anomalies in Nb and Ta in trace element diagrams. The prominent with strong positive anomalies in Pb (and U) suggest that the apparent trend is caused by some combination of alteration increase of U, contamination of Pb and analytical error.

Using solution ICP-MS, Pb analyses have the difficulty of Pb contamination of samples at all stages of the analytical process, as well as analytical uncertainties for samples with low Pb concentrations, or when special care is not taken with the analyses and the standards used. U analyses are problematic because even small amounts of alteration can increase the U contents of MORB, and depleted MORB have U concentrations near the detection limits of many laboratories, so that results less than 0.1 ppm are sometimes reported with only one significant figure. These problems lead to very scattered data and large ranges in these ratios. In many regional datasets, the relative variations in Ce/Pb or Nb/U are similar to La/Sm, even though variations in La/Sm are used as a general index of source enrichment and depletion.

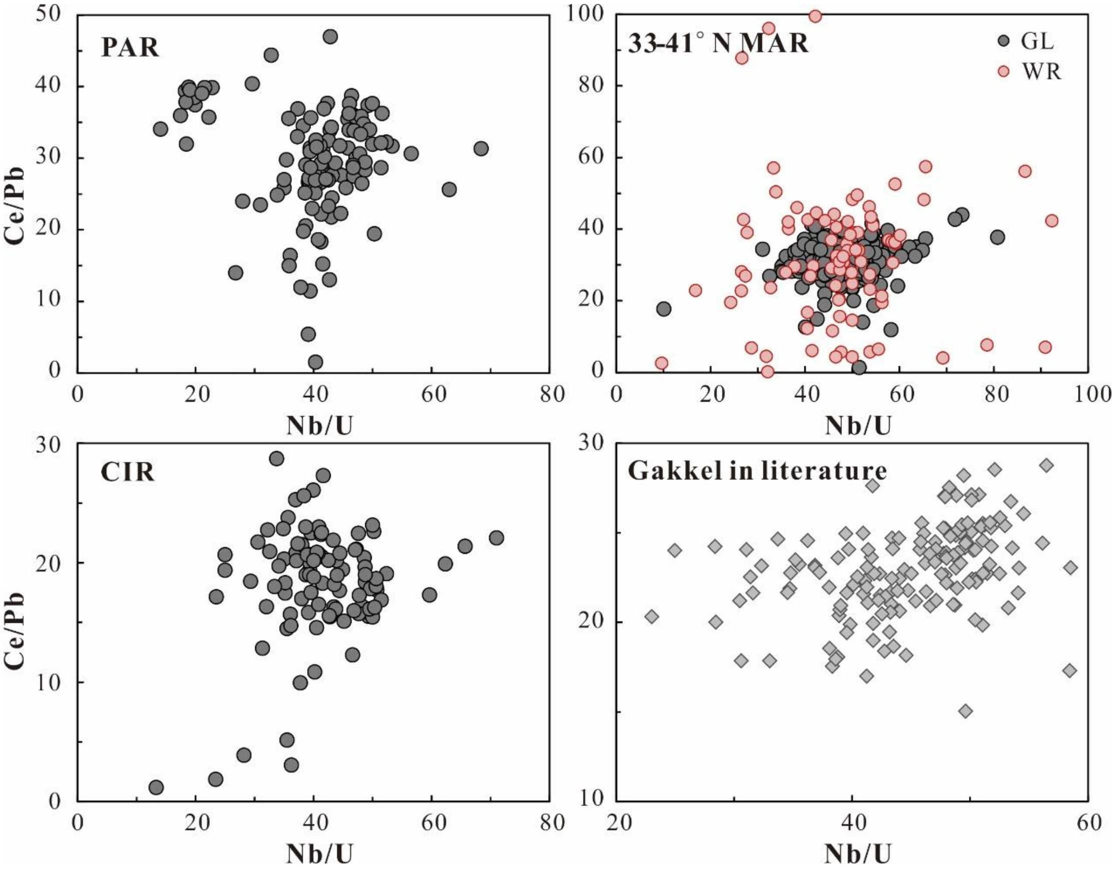

Supplementary Fig. 1 Nb/U versus Ce/Pb correlations of ICP-MS data for MORB from Pacific-Antarctic Ridge (PAR), 33-41°N Mid-Atlantic Ridge (MAR), Central Indian Ridge (CIR), and Gakkel MORB. Grey symbols are all glasses. All data were downloaded from PetDB.

Use of a laser ablation (LA)-ICP-MS to collect data avoids Pb contamination and U alteration, and the data quality of samples with low Pb and U concentrations can be largely improved by multiple analyses on the same batch of samples. Therefore, more consistent data can be acquired, as evidenced by new LA-ICP-MS data set of Gakkel MORB in this study, which lacks the extreme outliers, and begins to show a positive correlation between Nb/U and Ce/Pb (Supplementary Fig. 1b). Supplementary Fig. 2 shows the U and Pb contents of the same batch of Gakkel samples using both solution ICP-MS and LA-ICP-MS method. Many samples fall above the 1:1 line, indicating the solution ICP-MS U and Pb data show much larger uncertainty than laser data.

The consistency of data from multiple samples of the same dredge (Supplementary Fig. 1c) also increases confidence that the new LA-ICP-MS analyses have solved the problems of alteration and contamination, and that the ratios we measured are an accurate reflection of the magma. The consistency of the elemental ratios for restricted parts of the MOR, and their distinctiveness within regions increases our confidence that the ratios to construct the BABB filter reflect a mantle source signal, and are not related to shallower processes such as assimilation or interaction with the lower crust. The laser data presented in Supplementary Fig. 1 of the main text shows a good correlation between Ce/Pb and Nb/U that is absent in the older solution ICP-MS data presented in Supplementary Fig. 1d.

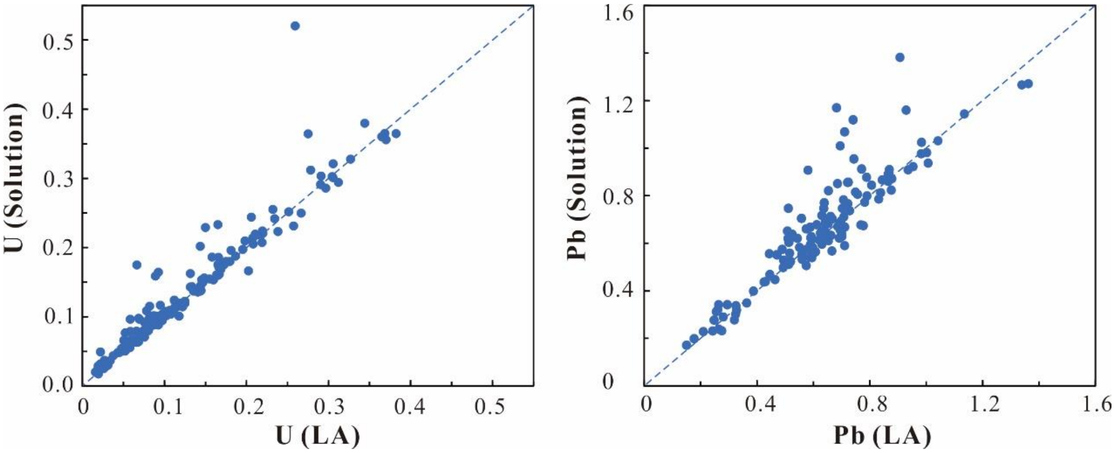

Supplementary Fig. 2 Comparison of U and Pb for solution ICP-MS data with *in situ* LA-ICP-MS data for Gakkel MORB. The diagonal lines are 1:1 correlation lines.

To circumvent these problems with most literature data, we identified some papers with clearly problematic Pb or U analyses, and eliminated those analyses from the data set. We also eliminated all values of Ce/Pb greater than 50.

In addition to the global data set of Gale et al[3], we added new papers which were included in PetDB, and did our own literature search to find additional papers not yet included in PetDB. After screening for papers without problematic Pb and U, the new data include data from Jenner and O'Neill[4] that were not included in the Gale et al.[3] compilation, Reekie et al.[5], Kelley et al.[6], Nielsen et al.[7], Jones et al.[8]; Clog[9], Standish[10], Gill, et al.[11], Janin et al.[12], Möller[13], Yang et al.[14], Hoernle et al.[15], Paquet, et al.[16], Mallick, et al.[17], Zong, et al.[18], Shimizu, et al.[19], Shimizu, et al.[20], and Escrig, Langmuir (unpublished), leading to 3000 more analyses than were included in Gale, et al.[3]. The new data compilation used for the global analysis that includes the original Gale et al.[3] file is given in its entirety in Data 6.

2. **Presentation of more detail on Gakkel Ridge**

The Gakkel ridge in the Arctic Ocean is the slowest spreading ridge on Earth, with a full spreading rate of ~15 mm/yr in the west near Lena Trough and as slow as ~7 mm/yr near the Siberian margin[21,22]. The 2001 Arctic Mid-Ocean Ridge Expedition (AMORE) sampled ~1000 km of the Gakkel Ridge between longitudes 7°W to 86°E.

We use a small number of elements to define the subduction influence because of the limitations of global data. This can be problematic because low Nb/U and Ce/Pb ratios are often observed in continental basalts, as basaltic magma erupted in continents may react with surrounding continental crust during its transportation through the continental lithosphere[23]. However, it is not the case for MORB. As the Gakkel Ridge is where new oceanic crust is produced, MORB magma would not be subject to continental crustal contamination. Therefore, the likely way to produce Nb depletion and Pb enrichment relative to U and Ce in fresh MORB glass is through subduction-derived contributions, where U and Pb are more mobile compared to Nb and Ce during slab dehydration, respectively[24]. Therefore, it appears that the BABB-like MORB from the Gakkel

Ridge, and the overall trend to lower Ce/Pb and Nb/U than the canonical values of these ratios are likely caused by contribution to the MORB source by materials affected by subduction processes.

This can be tested with other elements that are also sensitive to subduction processes. For example, Th/Nb and K_2O/TiO_2 ratios are also elevated by subduction. Plots of La/Sm vs. Th/Nb and K_2O/TiO_2 also shows many of the Gakkel samples offset in the direction of a subduction influence, as evident in the Pacific/BABB comparison for these ratios (Supplementary Fig. 3).

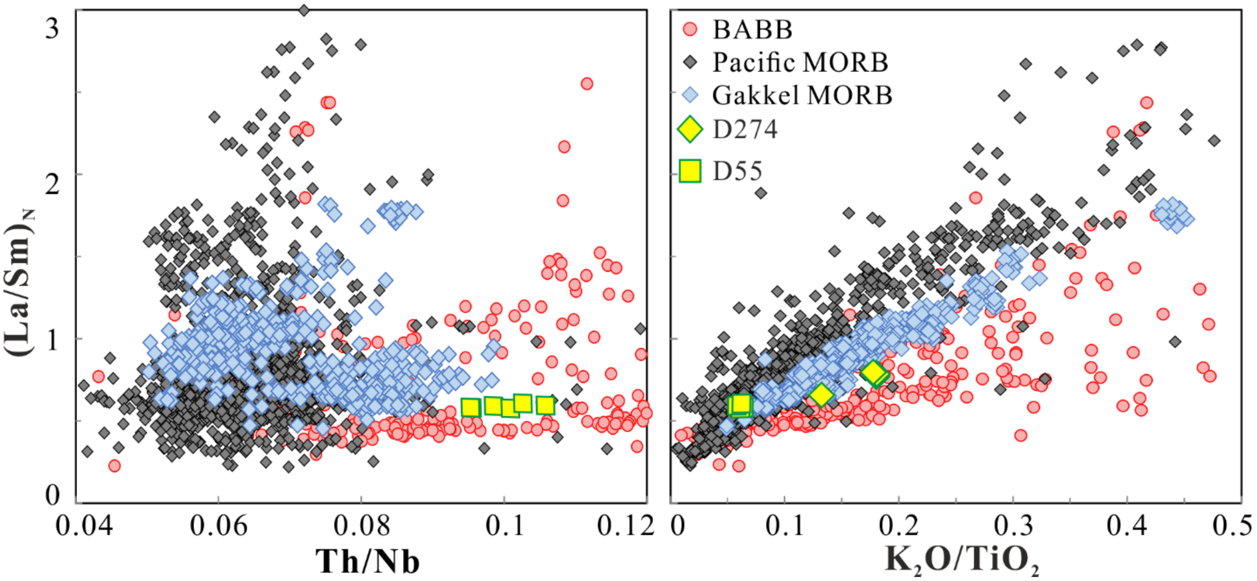

Supplementary Fig. 3 Th/Nb and K_2O/TiO_2 vs. $(La/Sm)_N$ for Pacific and Gakkel MORB as well as global BABB. Pacific MORB show clear separations with BABB with Th/Nb mostly lower than 0.08, independent of trace element enrichments indicated by $(La/Sm)_N$. MORB from Dredge 274 have high Th/Nb ratios up to 0.49, plotted way off the scale of diagram a). Significant amount of Gakkel MORB including Dredge 274 and 55 shift towards higher Th/Nb than 0.08, and higher K_2O/TiO_2 relative to $(La/Sm)_N$, similar to BABB, indicating a subduction influence.

These particular ratios are important because Goldstein et al[25] argued for the importance of a history involving phlogopite for some of the Gakkel samples with high Ba/Nb and Rb/Nb. But Th is not influenced by phlogopite[26], and therefore the offset in Th requires an additional explanation associated with subduction. The same reasoning applies to Ce/Pb and Nb/U ratios, which are also independent of phlogopite[26].

The tectonic history of the Arctic shows hundreds of millions of years of subduction from multiple directions towards this basin (Supplementary Fig. 4). The Gakkel Ridge is surrounded by continental lithosphere, and therefore spreading does not have open access to the surrounding mantle. This is a likely explanation for the strong subduction influence observed in this region.

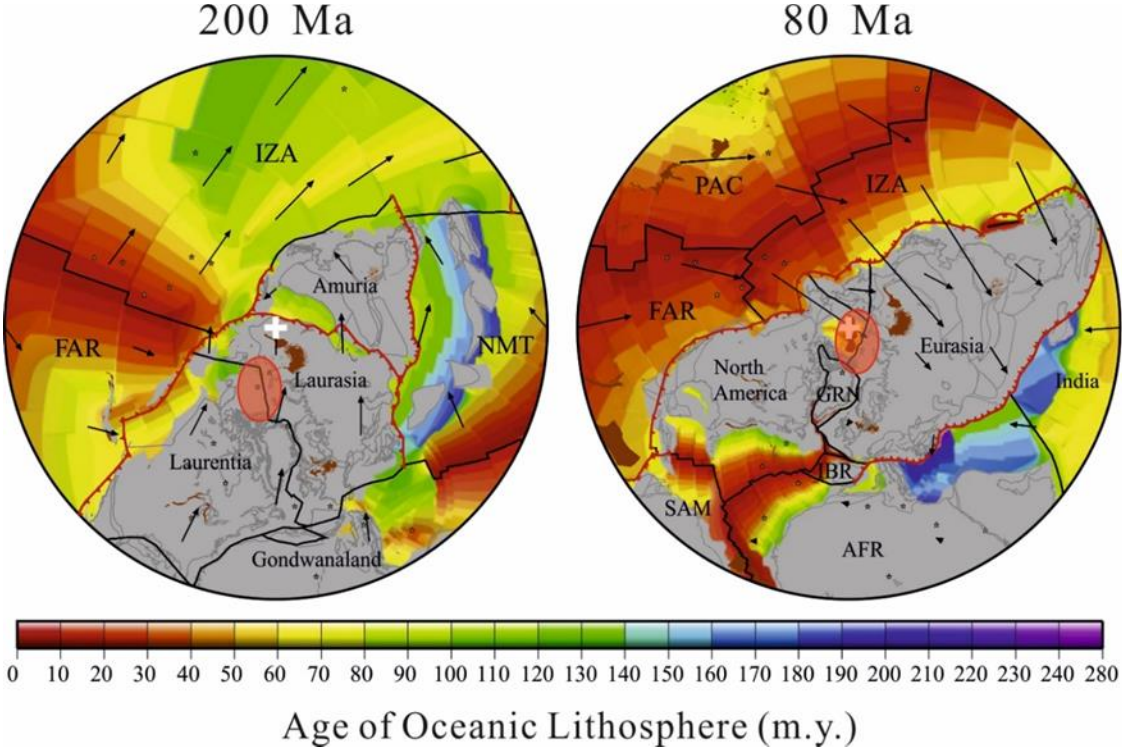

Supplementary Fig. 4 Continuous subduction events beneath Arctic Ocean shown by global plate reconstructions from North Pole (white crosses) projection at 200 Ma and 80 Ma, modified from Seton, et al.[27]. Red shaded fields represent the future location of Gakkel Ridge. Basemap shows the age–area distribution of oceanic lithosphere at the time of formation. Red lines denote subduction zones, black lines denote mid-ocean ridges and transform faults. Absolute plate velocity vectors are denoted as black arrows. Abbreviations for the plates are the same as in previous figures. Abbreviations include: AFR = African plate, FAR = Farallon plate, IBR = Iberian plate, IZA = Izanagi plate, NMT = North Meso-Tethys, PAC = Pacific plate, SAM = South American plate.

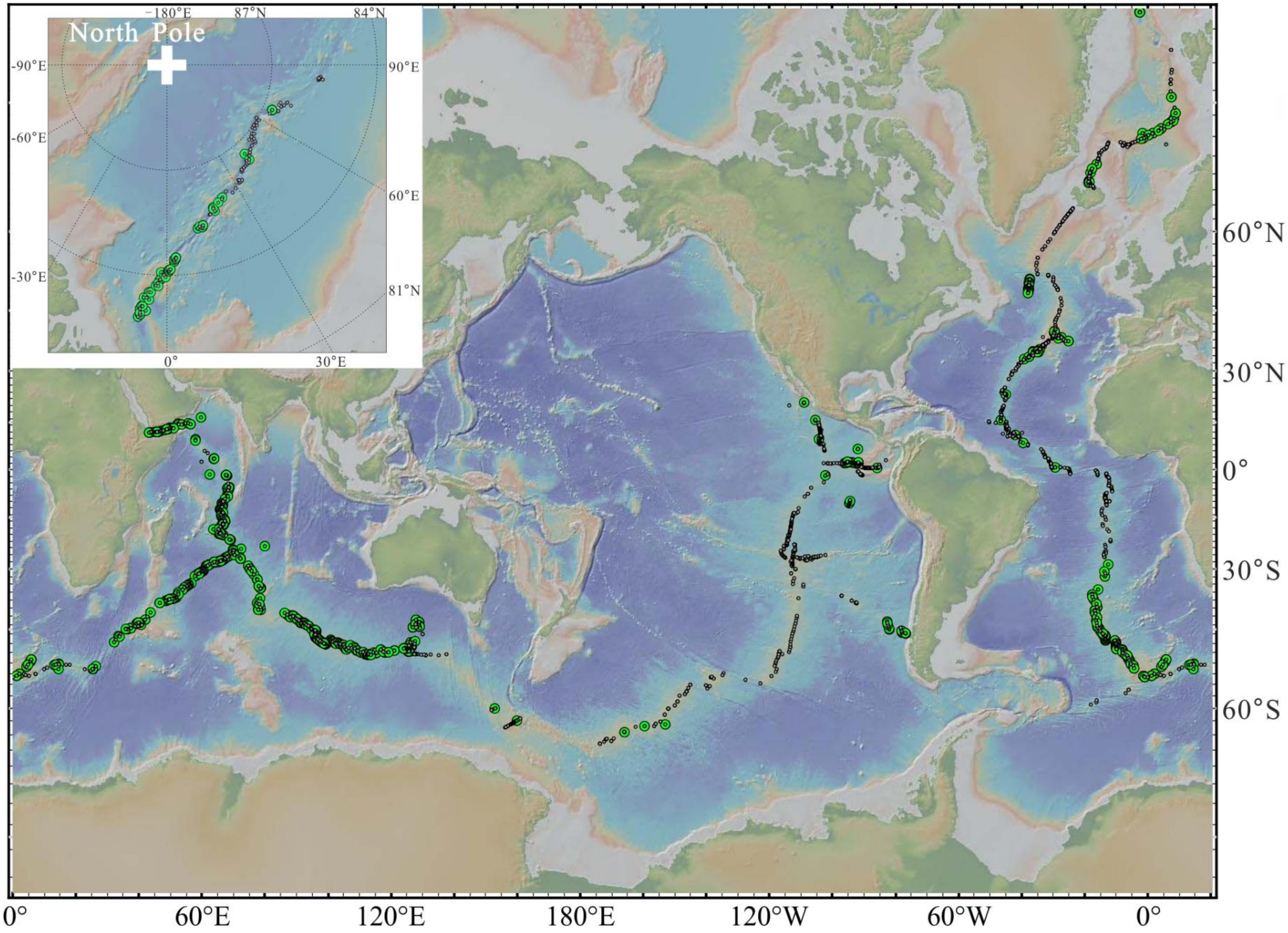

Supplementary Fig. 5 Distribution of MORB with DUPAL anomaly. Green dots are for global MORB samples with △8/4 > 30 (characteristic of DUPAL anomaly), and small grey dots are for all the samples with Pb isotopic data.

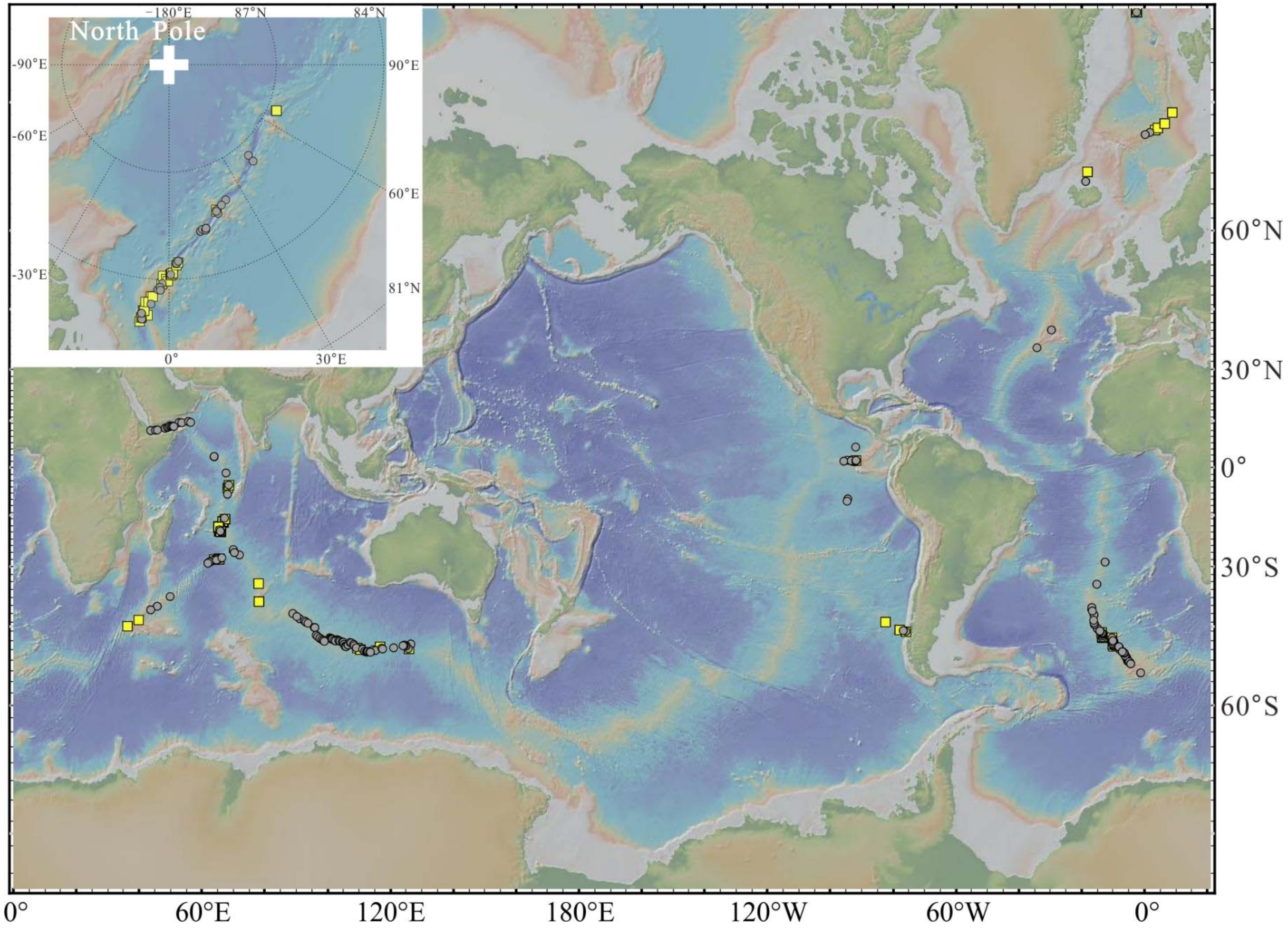

Supplementary Fig. 6 For samples with △8/4>30 and sufficient trace element data to evaluate the BABB filter, grey dots are locations that do not pass the filter while yellow squares show locations that do pass the filter.

Supplementary References

1 Hofmann, A. W., Class, C. & Goldstein, S. L. Size and composition of the residual and depleted mantle reservoir. *Preprint at https://www.essoar.org/doi/pdf/10.1002/essoar.10501102.1*, 43, doi:doi:10.1002/essoar.10501102.1 (2020).

2 Hofmann, A. W., Jochum, K., Seufert, M. & White, W. M. Nb and Pb in oceanic basalts: new constraints on mantle evolution. *Earth Planet. Sci. Lett.* **79**, 33-45 (1986).

3 Gale, A., Dalton, C. A., Langmuir, C. H., Su, Y. & Schilling, J.-G. The mean composition of ocean ridge basalts. *Geochem. Geophys. Geosyst.* **14**, 489-518, doi:10.1029/2012GC004334 (2013).

4 Jenner, F. E. & O'Neill, H. S. Analysis of 60 elements in 616 ocean floor basaltic glasses. *Geochemistry Geophysics Geosystems* **13**, doi:Q0200510.1029/2011gc004009 (2012).

5 Reekie, C. *et al.* Sulfide resorption during crustal ascent and degassing of oceanic plateau basalts. *Nature communications* **10**, 1-11 (2019).

6 Kelley, K. A., Kingsley, R. & Schilling, J.-G. Composition of plume-influenced mid-ocean ridge lavas and glasses from the Mid-Atlantic Ridge, East Pacific Rise, Galapagos Spreading Center, and Gulf of Aden. *Geochemistry Geophysics Geosystems* **14**, 223-242, doi:10.1029/2012gc004415 (2013).

7 Nielsen, S. G., Shimizu, N., Lee, C. T. A. & Behn, M. D. Chalcophile behavior of thallium during MORB melting and implications for the sulfur content of the mantle. *Geochem. Geophys. Geosyst.* **15**, 4905-4919 (2014).

8 Jones, M. *et al.* New constraints on mantle carbon from Mid-Atlantic Ridge popping rocks. *Earth Planet. Sci. Lett.* **511**, 67-75 (2019).

9 Clog, M. *Concentration et composition isotopique en hydrogène du manteau terrestre*, Paris 7, (2010).

10 Standish, J. J. The influence of ridge geometry at the ultraslow-spreading Southwest Indian Ridge (9°--25° E): Basalt composition sensitivity to variations in source and process. (2006).

11 Gill, J. *et al.* Spatial and Temporal Scale of Mantle Enrichment at the Endeavour Segment, Juan de Fuca Ridge. *J. Petrol.* **57**, 863-895, doi:10.1093/petrology/egw024 (2016).

12 Janin, M. *et al.* The Amsterdam-St. Paul Plateau: A complex hot spot/DUPAL-flavored MORB interaction. *Geochemistry Geophysics Geosystems* **13**, doi:10.1029/2012gc004165 (2012).

13 Möller, H. *Magma genesis and mantle sources at the Mid-Atlantic Ridge east of Ascension Island*, Christian-Albrechts Universität Kiel, (2002).

14 Yang, S., Humayun, M. & Salters, V. J. M. Elemental Systematics in MORB Glasses From the Mid-Atlantic Ridge. *Geochemistry Geophysics Geosystems* **19**, 4236-4259, doi:10.1029/2018gc007593 (2018).

15 Hoernle, K. *et al.* On- and off-axis chemical heterogeneities along the South Atlantic Mid-Ocean-Ridge (5-11 degrees S): Shallow or deep recycling of ocean crust and/or intraplate volcanism? *Earth Planet. Sci. Lett.* **306**, 86-97, doi:10.1016/j.epsl.2011.03.032 (2011).

16 Paquet, M., Cannat, M., Brunelli, D., Hamelin, C. & Humler, E. Effect of melt/mantle interactions on MORB chemistry at the easternmost Southwest Indian Ridge (61 degrees-67 degrees E). *Geochemistry Geophysics Geosystems* **17**, 4605-4640, doi:10.1002/2016gc006385 (2016).

17 Mallick, S., Salters, V. J. M. & Langmuir, C. H. Geochemical Variability Along the Northern East Pacific Rise: Coincident Source Composition and Ridge Segmentation. *Geochemistry Geophysics Geosystems* **20**, 1889-1911, doi:10.1029/2019gc008287 (2019).

18 Zong, T. *et al.* H2O in basaltic glasses from the slow-spreading Carlsberg Ridge: Implications for mantle source and magmatic processes. *Lithos* **332**, 274-286, doi:10.1016/j.lithos.2019.01.022 (2019).

19 Shimizu, K. *et al.* Two-component mantle melting-mixing model for the generation of mid-ocean ridge basalts: Implications for the volatile content of the Pacific upper mantle. *Geochim. Cosmochim. Acta* **176**, 44-80, doi:10.1016/j.gca.2015.10.033 (2016).

20 Shimizu, K. *et al.* Identifying volatile mantle trend with the water-fluorine-cerium systematics of basaltic glass. *Chem. Geol.* **522**, 283-294, doi:10.1016/j.chemgeo.2019.06.014 (2019).
21 Coakley, B. J. & Cochran, J. R. Gravity evidence of very thin crust at the Gakkel Ridge (Arctic Ocean). *Earth Planet. Sci. Lett.* **162**, 81-95, doi:10.1016/s0012-821x(98)00158-7 (1998).
22 Michael, P. *et al.* Magmatic and amagmatic seafloor generation at the ultraslow-spreading Gakkel ridge, Arctic Ocean. *Nature* **423**, 956-961 (2003).
23 DePaolo, D. & Wasserburg, G. Petrogenetic mixing models and Nd-Sr isotopic patterns. *Geochim. Cosmochim. Acta* **43**, 615-627 (1979).
24 Kessel, R., Schmidt, M. W., Ulmer, P. & Pettke, T. Trace element signature of subduction-zone fluids, melts and supercritical liquids at 120-180 km depth. *Nature* **437**, 724-727, doi:10.1038/nature03971 (2005).
25 Goldstein, S. L. *et al.* Origin of a 'Southern Hemisphere' geochemical signature in the Arctic upper mantle. *Nature* **453**, 89-93 (2008).
26 Schmidt, K. H., Bottazzi, P., Vannucci, R. & Mengel, K. Trace element partitioning between phlogopite, clinopyroxene and leucite lamproite melt. *Earth Planet. Sci. Lett.* **168**, 287-299, doi:10.1016/s0012-821x(99)00056-4 (1999).
27 Seton, M. *et al.* Global continental and ocean basin reconstructions since 200Ma. *Earth-Science Reviews* **113**, 212-270, doi:10.1016/j.earscirev.2012.03.002 (2012).
28 Turner, S. J. & Langmuir, C. H. The global chemical systematics of arc front stratovolcanoes: Evaluating the role of crustal processes. *Earth Planet. Sci. Lett.* **422**, 182-193, doi:10.1016/j.epsl.2015.03.056 (2015).

Supplementary Table 1 Chemical and isotopic compositions of MORB from Dredge 274 and 55, Gakkel Ridge

Sample	PS59-274-008	PS59-274-014	PS59-274-028	PS59-274-060	PS59-274-072	HLY0102-055-003	HLY0102-055-009	HLY0102-055-017	HLY0102-055-019	HLY0102-055-HE	HLY0102-055-SG
Dredge No.	274	274	274	274	274	55	55	55	55	55	55
Latitude (°N)	86.73	86.73	86.73	86.73	86.73	87.03	87.03	87.03	87.03	87.03	87.03
Longitude (°E)	66.87	66.87	66.87	66.87	66.87	59.47	59.47	59.47	59.47	59.47	59.47
Depth	-4567	-4567	-4567	-4567	-4567	-4174	-4174	-4174	-4174	-4174	-4174
Along-axis distance (k	807	807	807	807	807	753	753	753	753	753	753
SiO_2	49.39	50.33	50.37	50.36	50.16	49.27	48.82	49.09	48.33	48.33	48.66
TiO_2	1.02	1.06	1.03	1.05	1.04	1.02	1.04	1.03	1.04	1.05	1.02
Al_2O_3	17.25	17.11	17.19	17.05	17.17	17.22	17.73	17.29	17.85	17.81	17.66
FeO	9.46	8.86	8.71	8.87	8.80	9.66	9.48	9.55	9.59	9.64	9.56
MnO	0.17	0.17	0.16	0.16	0.16	0.17	0.17	0.17	0.18	0.17	0.17
MgO	8.80	8.28	8.45	8.33	8.40	8.54	8.58	8.97	8.74	8.79	8.87
CaO	10.28	10.62	10.56	10.59	10.71	10.60	10.72	10.49	10.83	10.81	10.69
Na_2O	3.12	2.98	2.96	3.01	3.01	3.06	3.00	2.96	3.00	2.98	2.95
K_2O	0.14	0.19	0.19	0.19	0.18	0.07	0.06	0.06	0.06	0.06	0.06
P_2O_5	0.07	0.07	0.06	0.07	0.07	0.08	0.08	0.07	0.08	0.08	0.08
total	99.69	99.67	99.69	99.70	99.70	99.68	99.69	99.70	99.69	99.72	99.72
H_2O	0.247	0.226	0.234	0.241	0.230				0.288		
Li	6.27	6.68	6.46	6.82	6.52	5.48	4.31	4.47	4.84	3.51	4.22
B	1.68	1.91	1.90	2.38	2.26	0.662	0.410	0.920	0.977	0.670	0.907
Be	0.441	0.470	0.465	0.425	0.432	0.296	0.501	0.447	0.445	0.484	0.368
Sc	34.7	35.6	34.8	34.9	35.4	39.2	40.5	38.1	39.9	40.1	40.9
V	170	188	181	190	187	168	170	165	168	170	169
Cr	312	300	291	303	302	281	267	280	291	290	286
Co	46.3	43.5	42.8	44.0	44.4	46.5	45.9	46.8	45.9	49.1	47.5
Ni	156	120	128	125	132	144	141	157	146	171	157
Cu	93	87.0	86.7	91.3	92.4	97	92.1	97.2	96	108.0	94.9
Zn	66.6	61.3	60.9	63.0	62.4	58.8	61.1	60.2	61.3	63.4	67.7
Ga	15.6	15.1	14.9	15.4	15.6	15.2	15.0	14.3	14.6	15.2	14.7
Ge	1.33	1.31	1.21	1.27	1.27	1.38	1.40	1.33	1.42	1.30	1.34
Rb	3.54	5.77	5.46	5.71	5.60	0.826	0.789	0.822	0.792	0.844	0.825
Sr	130	111	110	111	112	139	144	141	146	149	144
Y	24.8	25.3	24.2	24.6	24.8	26.6	28.2	26.7	27.6	28.1	28.4
Zr	65.1	76.8	73.7	75.3	74.9	67.9	71.2	68.3	70.2	70.9	71.7
Nb	0.90	1.11	1.06	1.10	1.07	1.01	1.04	1.02	1.01	1.06	1.01
Mo	0.160	0.135	0.148	0.173	0.147	0.146	0.170		0.162	0.166	0.202
Ag	0.032	0.037	0.034	0.047	0.035	0.070	0.024	0.030	0.050	0.019	0.024
Cd	0.128	0.129	0.115	0.121	0.141	0.062	0.150	0.096	0.136	0.151	0.148
In	0.060	0.055	0.056	0.067	0.068	0.061	0.066	0.063	0.069	0.062	0.064
Sn	0.709	0.714	0.686	0.742	0.773	0.747	0.681	0.725	0.681	0.741	0.684
Sb	0.018	0.027	0.025	0.028	0.021	0.004	0.012	0.013	0.001	0.004	0.008
Cs	0.158	0.237	0.220	0.249	0.239	0.031	0.026	0.033	0.029	0.030	0.030
Ba	22.4	38.5	37.0	38.7	37.2	5.70	5.67	5.82	5.93	5.78	5.88
La	2.51	3.26	3.10	3.22	3.15	2.11	2.16	2.11	2.08	2.16	2.15
Ce	7.75	9.18	8.74	9.23	9.01	6.71	7.01	6.72	6.94	7.03	6.78
Pr	1.29	1.47	1.42	1.50	1.44	1.14	1.17	1.13	1.18	1.15	1.17
Nd	6.93	7.79	7.48	7.69	7.63	6.40	6.57	6.19	6.37	6.45	6.28
Sm	2.39	2.65	2.49	2.56	2.47	2.28	2.33	2.23	2.26	2.28	2.22
Eu	0.942	0.99	0.96	0.97	0.98	0.926	0.933	0.894	0.901	0.963	0.934
Gd	3.169	3.378	3.275	3.328	3.374	3.153	3.195	3.072	3.242	3.229	3.174
Tb	0.60	0.62	0.60	0.61	0.61	0.58	0.62	0.59	0.62	0.63	0.63
Dy	4.01	4.15	3.98	4.03	4.13	4.14	4.42	4.24	4.32	4.43	4.36
Ho	0.878	0.901	0.877	0.882	0.889	0.962	1.008	0.949	0.99	0.99	1.00
Er	2.56	2.58	2.48	2.49	2.48	2.82	3.06	2.83	2.85	2.95	2.96
Tm	0.376	0.403	0.376	0.368	0.378	0.440	0.463	0.429	0.459	0.454	0.437
Yb	2.47	2.50	2.39	2.44	2.49	2.97	3.02	2.89	3.05	3.12	2.98
Lu	0.397	0.385	0.376	0.370	0.375	0.450	0.490	0.446	0.452	0.452	0.469
Hf	1.72	2.02	1.95	1.94	1.95	1.72	1.72	1.65	1.62	1.68	1.69
Ta	0.063	0.081	0.076	0.074	0.076	0.077	0.074	0.072	0.073	0.068	0.073
W	0.039	0.086	0.074	0.089	0.082	0.019	0.018	0.019	0.015	0.014	0.018
Tl	0.026	0.038	0.032	0.037	0.033	0.009	0.018	0.013	0.015	0.013	0.018
Pb	0.99	1.28	1.29	1.37	1.28	0.417	0.446	0.426	0.464	0.426	0.430
Th	0.318	0.520	0.502	0.510	0.490	0.097	0.099	0.100	0.102	0.112	0.104
U	0.087	0.139	0.134	0.141	0.131	0.039	0.042	0.043	0.045	0.041	0.038
Nb/U	10.4	8.0	7.9	7.8	8.1	26.1	24.8	23.9	22.4	25.9	26.9
Ce/Pb	7.8	7.1	6.8	6.7	7.1	16.1	15.7	15.8	14.9	16.5	15.8
H_2O/Ce	319	247	268	261	255				415		
$^{87}Sr/^{86}Sr$	0.703577	0.703740	0.703782	0.703728	0.703705	0.702894	0.702889		0.702874		
2SE combined error	0.000021	0.000022	0.000021	0.000018	0.000020	0.000009	0.000009		0.000009		
$^{143}Nd/^{144}Nd$	0.512887	0.512870	0.512883	0.512885	0.512874	0.513099	0.513078		0.513102		
2SE combined error	0.000016	0.000019	0.000019	0.000015	0.000018	0.000020	0.000013		0.000013		
$^{206}Pb/^{204}Pb$	18.267	18.480	18.471	18.469	18.470	18.327	18.312		18.321		
2SE combined error	0.001	0.001	0.001	0.001	0.001	0.001	0.003		0.003		
$^{207}Pb/^{204}Pb$	15.529	15.550	15.539	15.538	15.540	15.474	15.465		15.465		
2SE combined error	0.001	0.001	0.001	0.001	0.001	0.001	0.004		0.004		
$^{208}Pb/^{204}Pb$	38.109	38.410	38.388	38.384	38.410	37.986	37.952		37.959		
2SE combined error	0.003	0.002	0.002	0.003	0.002	0.003	0.007		0.007		

Note: 2SE error reported for isotope ratios are calculated as the square roots of the sum of the squares of 2SE internal error and the 2SD external error.

Supplementary Table 2. Average Pacific, BABB-like MORB and arc volcanics compositions used in the model

	melt volume	Cs	Rb	Ba	U	Th	K	Nb	f_{Cs}	f_{Rb}	f_{Ba}	f_U	f_{Th}	f_K
	km^3/yr	ppm							$km^3/yr*ppm$					
Pacific MORB average		0.023	1.75	17.9	0.088	0.251	1184	4.09						
BABB-like MORB average	2.73	0.050	3.96	43.9	0.124	0.416	1884	4.96	0.06	5.02	60.7	0.05	0.31	1222
BABB average	1.30	0.129	6.16	64.3	0.161	0.440	1959	3.01	0.15	6.34	66.5	0.13	0.33	1415
Arc volcanics average	2.00	0.480	12.4	225	0.413	1.02	5476	1.71	0.94	23.2	436	0.75	1.82	9961

	MgO	Cs	Rb	Ba	Th	U	Nb	Ta	K	La	H_2O[b]	Ce	Pb	Pr	Sr	Nd	Zr	Hf	Sm	Eu	Ti	Gd	H_2O/Ce
Pacific log-normal mean>4	7.45	0.023	1.75	17.9	0.251	0.088	4.09	0.267	1184	4.45	0.263	12.8	0.505	2.09	126	11.0	103	2.64	3.64	1.31	9683	4.77	205
BABB-like MORB log-normal me	7.43	0.050	3.96	43.9	0.416	0.124	4.96	0.306	1884	5.41	0.420	14.3	0.737	2.15	152	11.3	101	2.46	3.51	1.27	8884	4.47	293
enrichment factor[a]		2.16	2.27	2.46	1.66	1.41	1.21	1.15	1.59	1.21	1.60	1.12	1.46	1.03	1.20	1.03	0.97	0.93	0.97	0.97	0.92	0.94	

note:

Compositions for Pacific MORB and BABB-like MORB averages are log-normal means for all Pacific MORB with MgO>4 wt.% and BABB-like MORB compiled in this study.

Compositions for BABB averages are mean BABB data from Gale et al.[3]. Compositions for arc volcanics are log-normal means for global arc averages at MgO of 6 wt% in Turner and Langmuir[28].

Calculation on subduction flux f for Rb, Ba, U, Th, and K can be found in the main context.

[a]Enrichment factors of BABB-like MORB relative to Pacific MORB, calculated by dividing BABB-like MORB log-normal means by Pacific MORB log-normal means.

[b]H_2O estimated from average H_2O/Ce ratio multiplied by average Ce content.
