# Supplementary material for: A subduction influence on ocean ridge basalts outside the Pacific subduction shield
Source: Nat Commun. 2021 Aug 6;12:4757. doi: 10.1038/s41467-021-25027-2 (PMC8346520; doi:10.1038/s41467-021-25027-2)
Supplement: Supplementary file 2 — Description of Additional Supplementary Files [file 41467_2021_25027_MOESM2_ESM.docx]

Description of Additional Supplementary Files

File name: Supplementary Discussion and Supplementary Figures 1-6

Description: Analytical problems with U and Pb in literature MORB dataset, presentation of more detail on Gakkel Ridge and distribution of MORB with Dupal anomaly.

File name: Supplementary Table 1

Description: Chemical and isotopic compositions for MORB from Dredge 274 and 55

File name: Supplementary Table 2

Description: Average Pacific, BABB-like MORB, BABB and arc volcanics compositions used in the slab flux calculation

File name: Supplementary Data 1

Description: Major and trace element compositions for the in-house quality control standard VE32 and BHVO-2G for standardization

File name: Supplementary Data 2

Description: LA-ICP-MS measurement on K_2_O, TiO_2_, Ba, La, Ce, Sr, Nd, Sm, Nb, Pb, Rb, Th and U, and FTIR measurement on H_2_O contents for Gakkel MORB

File name: Supplementary Data 3

Description: Rb/Nb, Ba/Nb, Nb/U, Ce/Pb, Th/Nb and H_2_O/Ce ratios for Gakkel samples averaged per dredging station

File name: Supplementary Data 4

Description: Data compilation for global mid-ocean ridge and back-arc basin basalts
